# Supplementary material for: MK2 Inhibition Induces p53-Dependent Senescence in Glioblastoma Cells
Source: Cancers (Basel). 2020 Mar 11;12(3):654. doi: 10.3390/cancers12030654 (PMC7139970; doi:10.3390/cancers12030654)
Supplement: Supplementary file 1 [file cancers-12-00654-s001.pdf]

A

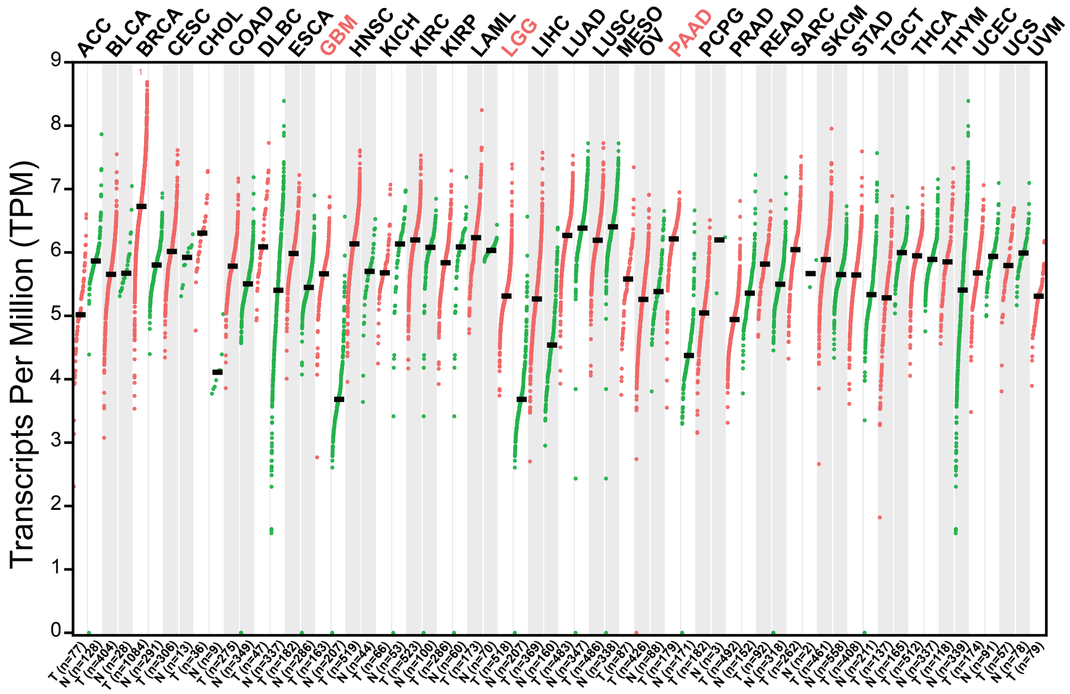

B

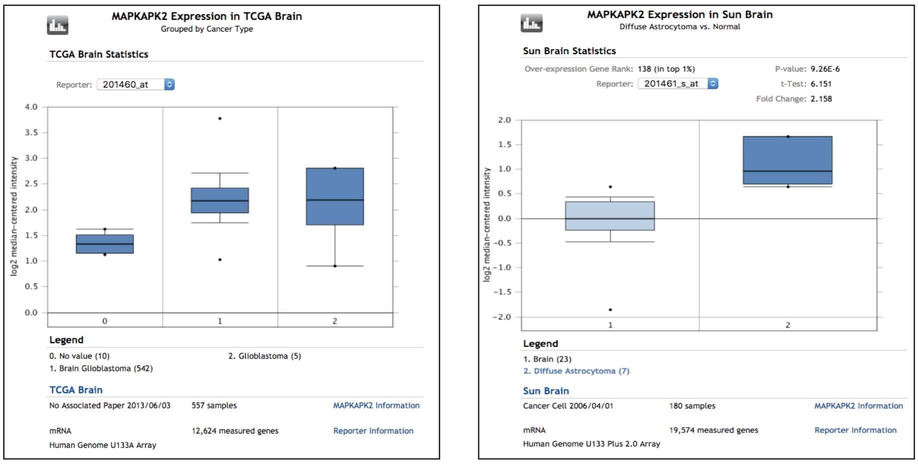

C

	MK2 positive	MK2 negative	P value	p-MK2 positive	p-MK2 negative	P value
Gender (female)	6	14		33	16	
Gender (male)	17	23	P = 0.348	4	4	P = 0.340
Age (mean, median, SD)	50,52,16	47,51,17		53,54,12	47,51,16	
De novo GBM	10	22		6	24	
Secondary GBM	8	2	P = 0.009	2	8	P = 1.000
IDH1 positive	10	5		2	12	
IDH1 negative	15	35	P = 0.013	8	40	P = 0.831
Glioma grade I	4	11		3	12	
Glioma grade II	11	17		1	24	
Glioma grade III	8	9		0	15	
Glioma grade IV	26	40	P = 0.699	10	53	P = 0.144
Primary surgery (GBM)	9	20		6	21	
2nd surgery (GBM)	12	18		2	27	
3rd surgery (GBM)	5	1		1	5	
4th surgery (GBM)	0	1	P = 0.096	1	0	P = 0.049

Supplementary Figure S1. (A) Profile of MAPKAPK-2 (MK2) mRNA expressed as transcripts per million across 33 different cancer types. MAPKAPK-2 mRNA levels are normalised to normal tissue controls per group. Data acquired from GEPIA (http://gepia.cancer-pku.cn) (B) MK2 gene analysis of TCGA and Sun Brain databases using Oncomine (www.oncomine.org). (C) Clinical data for formalin-fixed, paraffin embedded tissues from 60 brain tumour patients (used for immunohistochemistry in Fig. 1F and 1G) diagnosed with grade I-IV astrocytomas who underwent surgery in the Unit of Neurosurgery, Tampere University Hospital, Tampere, Finland during 1983-2005. P values were calculated using chi-square test except for age which was calculated using the Mann-Whitney test.

Supplementary Figure S2

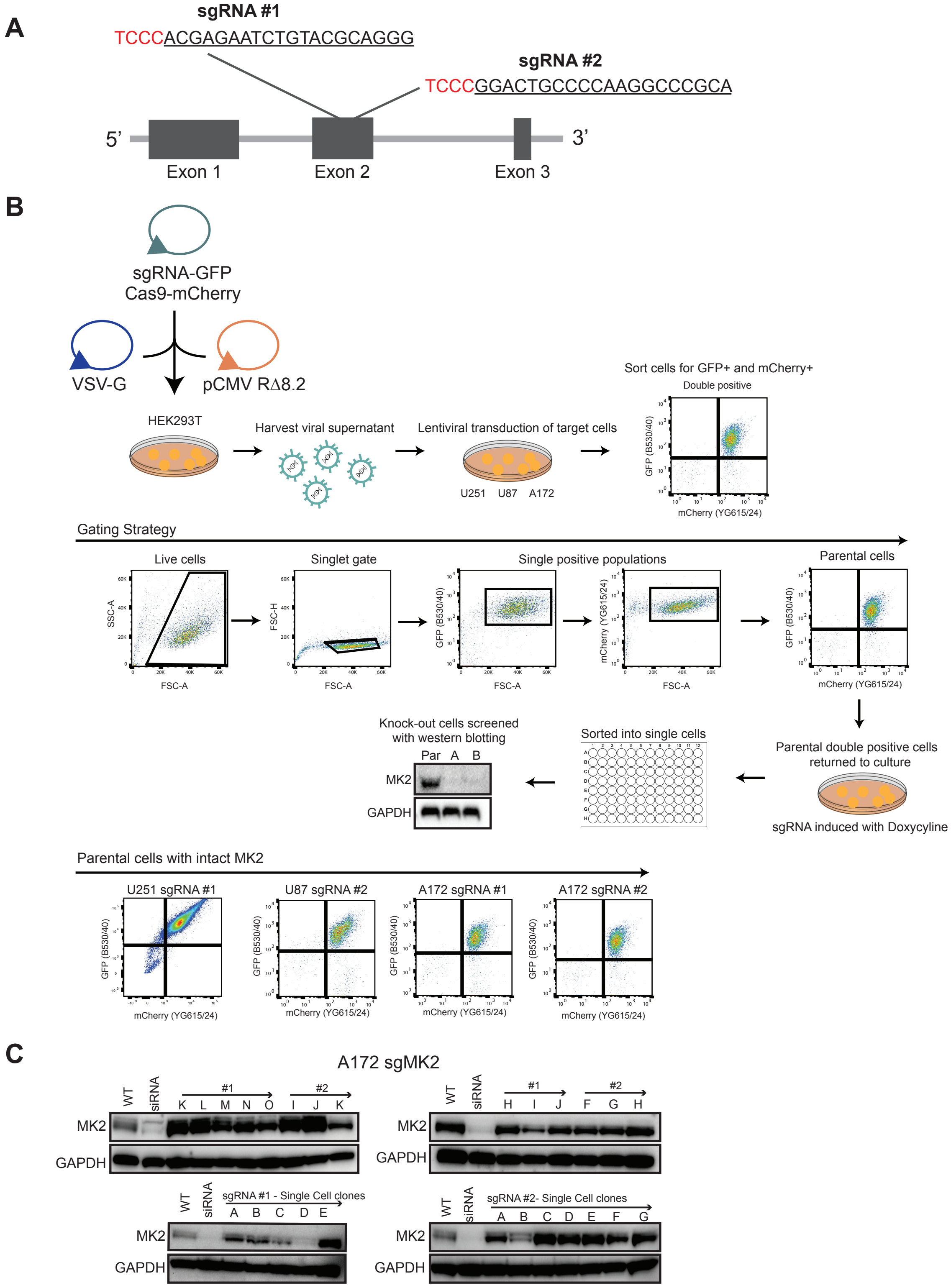

Supplementary Figure S2. Schematic overview of the CRISPR-Cas9 gene editing to knock-out MK2 in glioblastoma cells. (A) Schematic diagram of the germline configuration of the human MAPKAPK-2 (MK2) gene. The target sites of sgRNAs are indicated. (B) Schematic diagram of the CRISPR/Cas9 infection, gating strategy and monoclonal isolation of infected A172, U87 and U251 cells. (C) Western blot screen for $MK2^{null}$ A172 monoclonal cell lines. MK2 protein was detected in all 26 single cell

Supplementary Figure S3

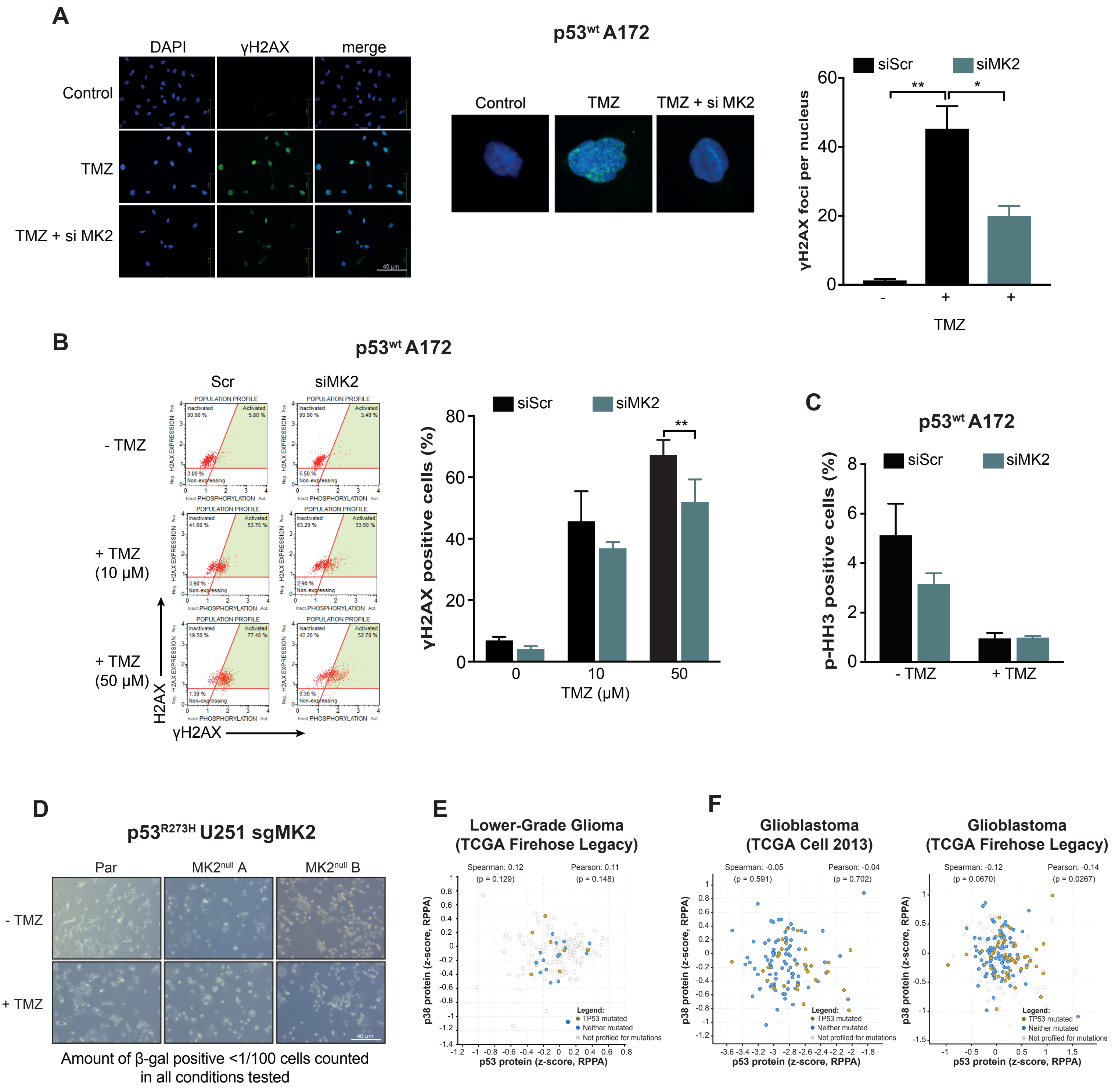

Supplementary Figure S3. (A) Immunofluorescence and quantification of A172 cells treated with siMK2 and temozolomide (TMZ; 50 μM, 72h), cells were stained with Alexa488-labelled anti-γH2AX (green) and DAPI (blue). Representative images of three independent experiments are shown. Graph bars represent mean ± SEM. (* $P < 0.05$, ** $P < 0.01$, two-way ANOVA followed by Bonferroni post-test) (B) A172 cells transfected and treated as in (A) were double stained with Alexa555-labelled anti-phospho H2AX Ser139 (γH2AX) and an anti-H2AX PECy5 conjugated antibody using the Muse H2A.X Activation Dual Detection Kit (#MCH200101, Merck Millipore). Samples were analysed using a Muse Cell analyzer (Merck Millipore). Representative FACS plots for each condition are shown. Graph bars represent mean ± SEM from three independent experiements. (** $P < 0.01$, two-way ANOVA followed by Bonferroni post-test). (C) Quantification of the mitotic entry marker p-HH3 in A172 cells treated with siMK2 and temozolomide (TMZ; 50 μM) using flow cytometry. Graph bars represent mean ± SEM of three independent experiments. (D) Senescence associated β-galactosidase (β-gal) staining of parental and p53^{R273H} U251 cells treated with temozolomide (TMZ; 25 μM, 10 days). Representative images of three independent experiments are shown. (E-F) Pearson and Spearman correlations of p53 and p38 MAPK (MAPK14) protein expression in the TCGA Provisional dataset for lower-grade glioma and the TCGA Cell 2013 dataset for glioblastoma (cbioportal.org).

Supplementary Figure S4

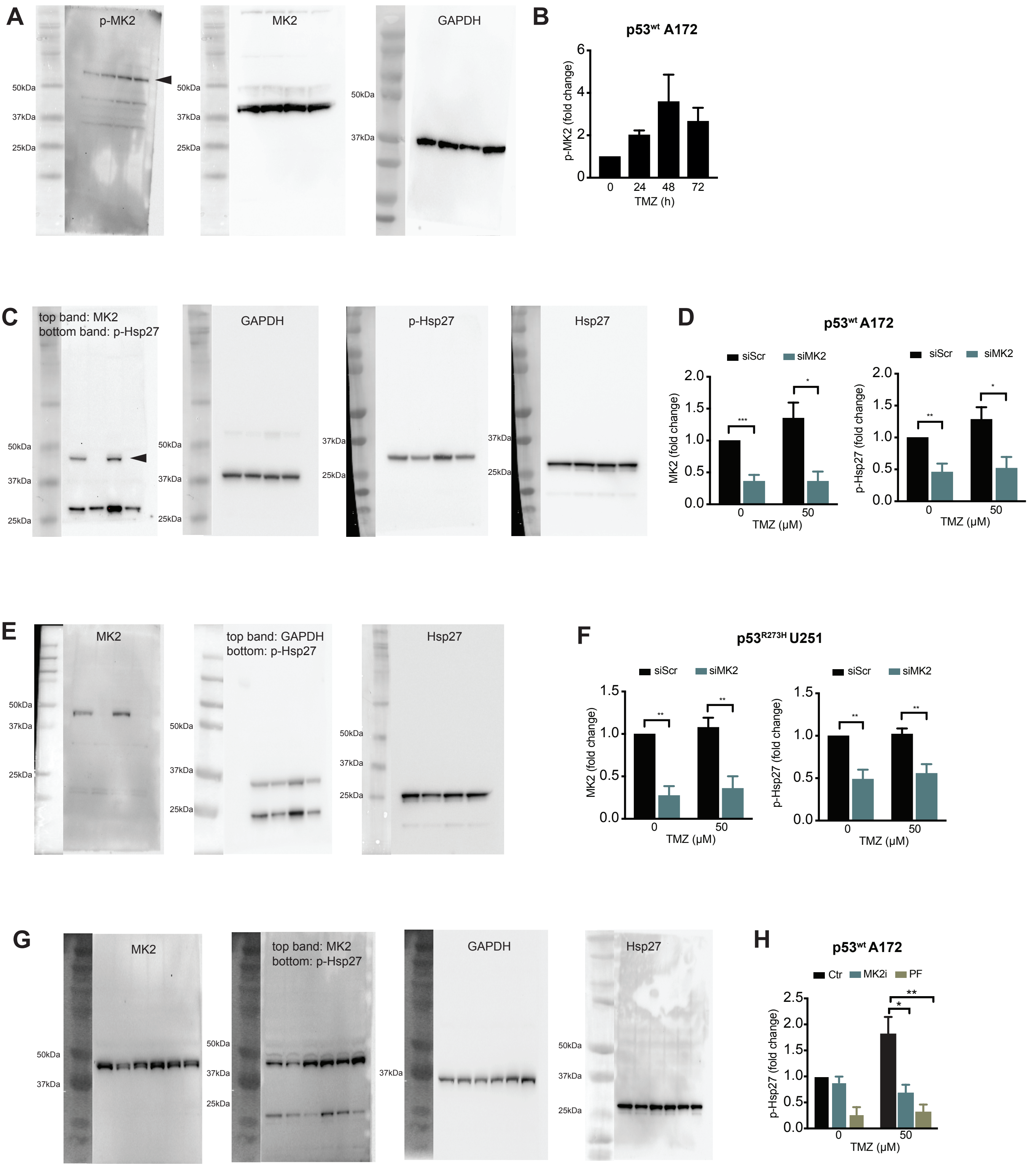

Supplementary Figure S4

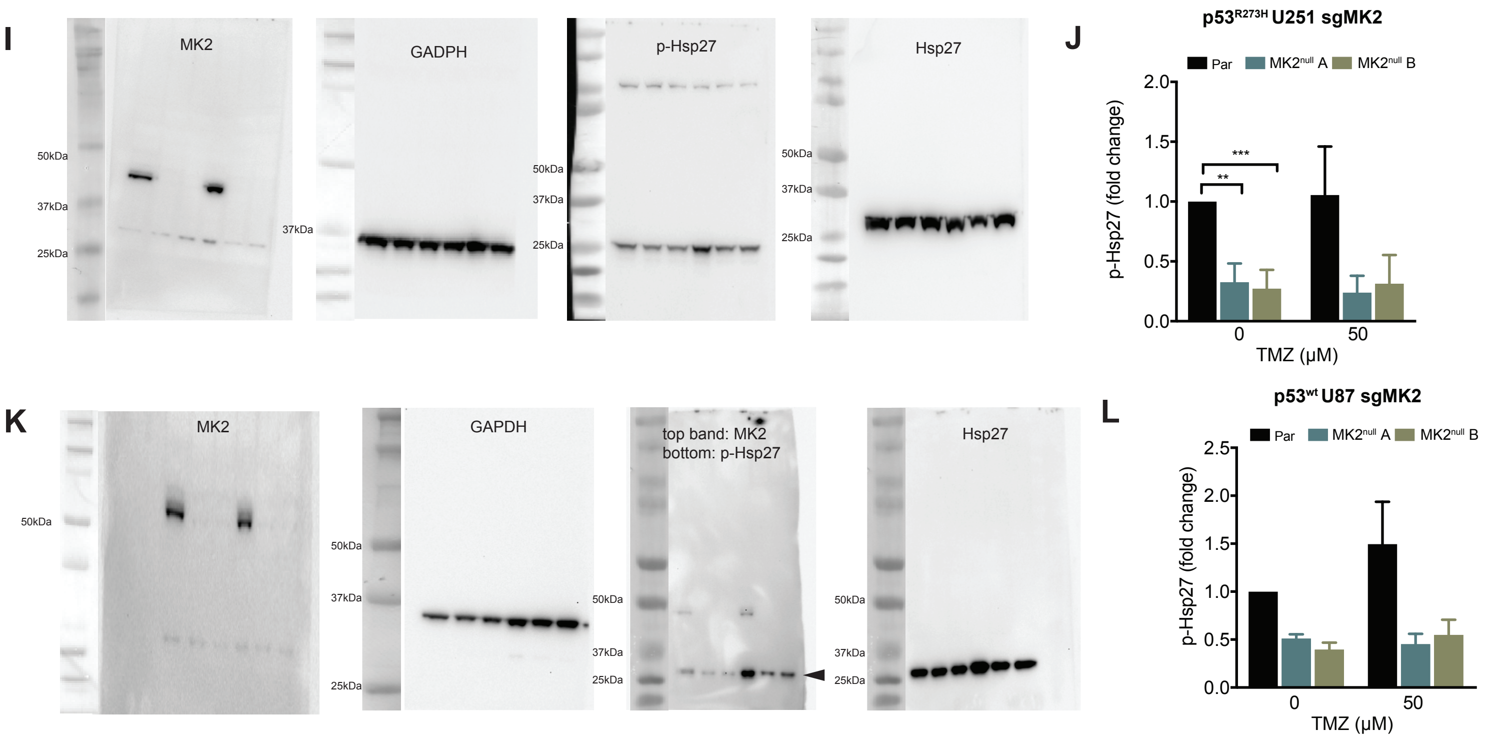

Supplementary Figure S4.

A) Supplementary data Western blot gel images corresponding to Figure 2A.
B) Graph represents p-MK2 levels of n=3 corresponding to Figure 2A.
C) Supplementary data Western blot gel images corresponding to Figure 2B.
D) Graphs represent MK2 and p-Hsp27 levels of n=4 corresponding to Figure 2B (t-test, $*P < 0.05$; $**P < 0.01$; $*** P < 0.001$).
E) Supplementary data Western blot gel images corresponding to Figure 2C.
F) Graphs represent MK2 and p-Hsp27 levels of n=4 corresponding to Figure 2C (t-test, t-test, $** P < 0.01$).
G) Supplementary data Western blot gel images corresponding to Figure 2F.
H) Graph represents p-Hsp27 levels of n=3 corresponding to Figure 2F (t-test, $*P < 0.05$; $**P < 0.01$).
I) Supplementary data Western blot gel images corresponding to Figure 2I.
J) Graph represents MK2 and p-Hsp27 levels of n=4 corresponding to Figure 2I (t-test, $**P < 0.01$; $***P < 0.001$).
K) Supplementary data Western blot gel images corresponding to Figure 2J.
L) Graph represents MK2 and p-Hsp27 levels of n=3 corresponding to Figure 2J.

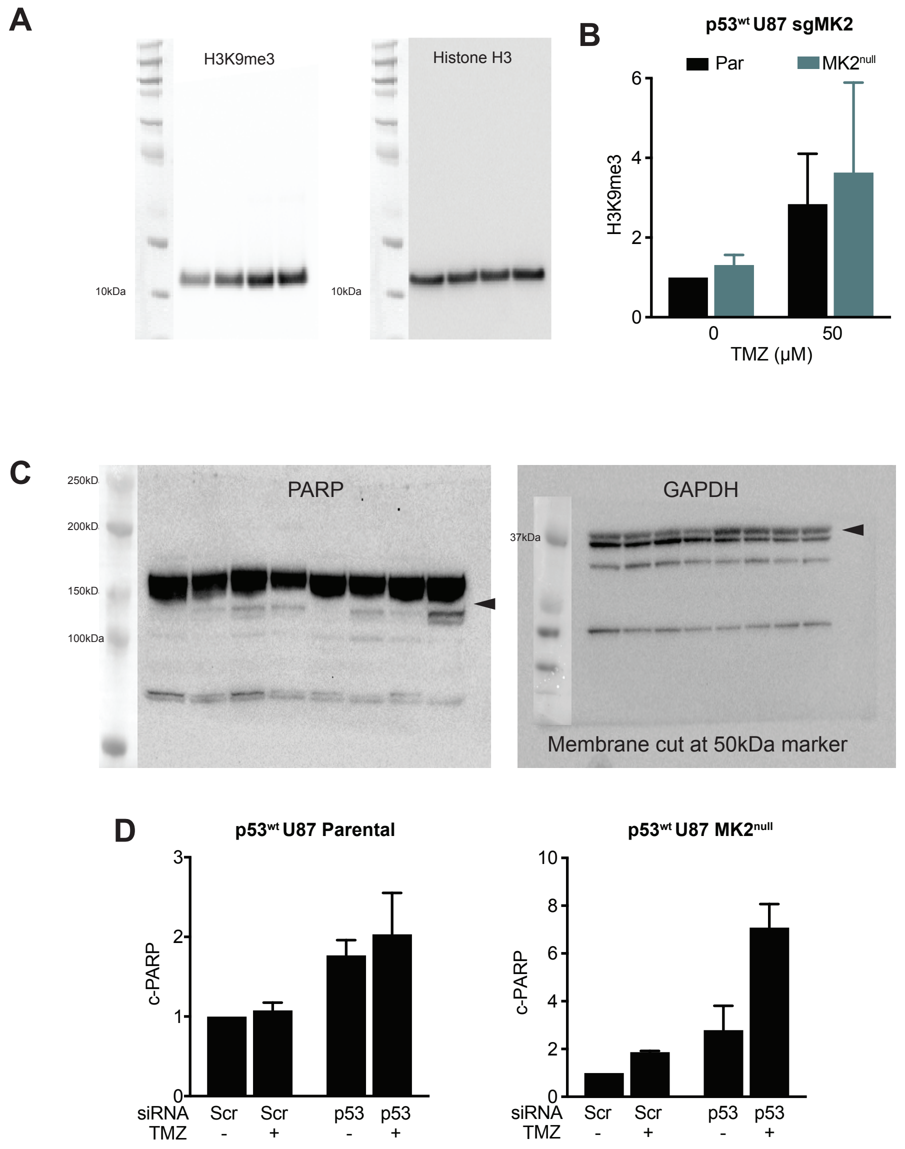

Supplementary Figure S5.

A) Supplementary data Western blot gel images corresponding to Figure 3H.

B) Graph represents H3K9me3 levels of n=3 corresponding to Figure 3H.

C) Supplementary data Western blot gel images corresponding to Figures 3L and 3M.

D) Graphs represent c-PARP levels of n=3 corresponding to Figures 3L and 3M.

Supplementary Figure S6

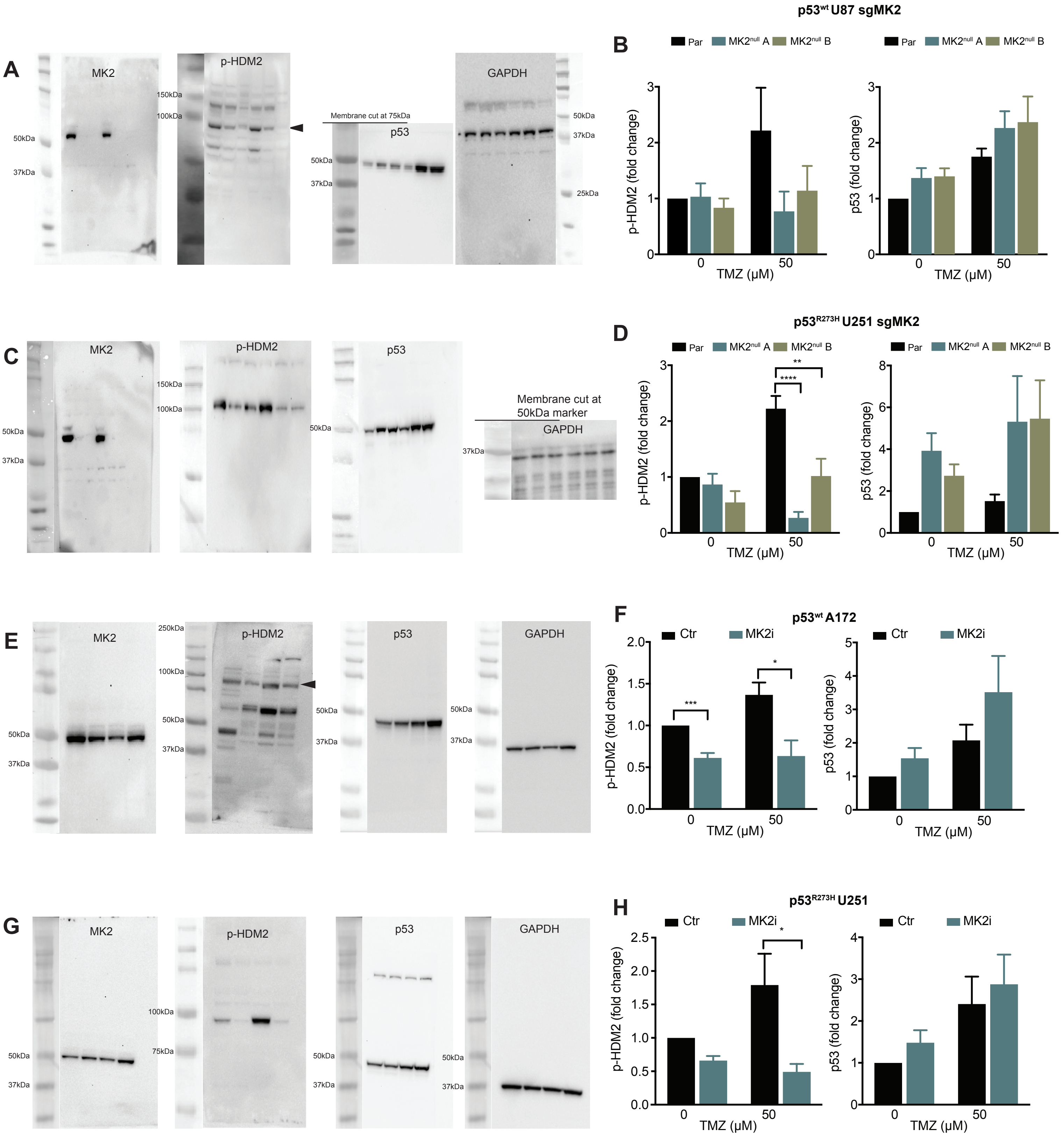

Supplementary Figure S6

M

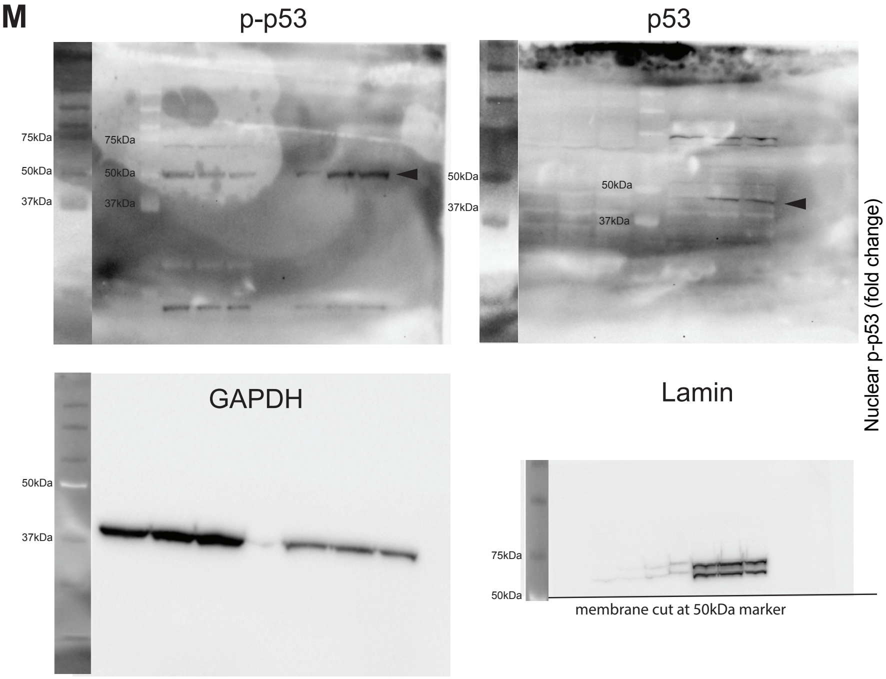

N

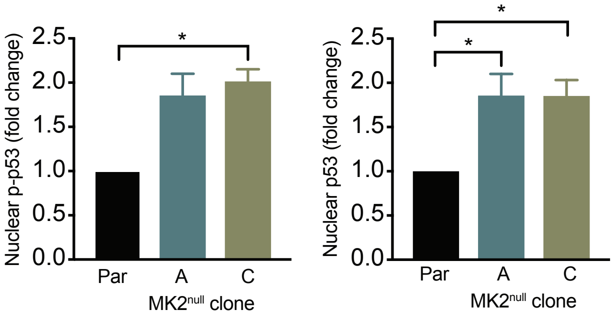

Supplementary Figure S6.

A) Supplementary data Western blot gel images corresponding to Figure 4A.
B) Graphs represent p-HDM2 and p53 levels of n=4 corresponding to Figure 4A.
C) Supplementary data Western blot gel images corresponding to Figure 4B.
D) Graphs represent p-HDM2 and p53 levels of n=4 corresponding to Figure 4B (t-test, **$P < 0.01$; ****$P < 0.0001$).
E) Supplementary data Western blot gel images corresponding to Figure 4C.
F) Graphs represent p-HDM2 and p53 levels of n=4 corresponding to Figure 4C (t-test, *$P < 0.05$; ***$P < 0.01$).
G) Supplementary data Western blot gel images corresponding to Figure 4D.
H) Graphs represent p-HDM2 and p53 levels of n=4 corresponding to Figure 4D (t-test, *$P < 0.05$).
I) Supplementary data Western blot gel images corresponding to Figure 4E.
J) Graphs represent p53 half-life curve of n=3 corresponding to Figure 4E.
K) Supplementary data Western blot gel images corresponding to Figure 4G.
L) Graphs represent p-p53 and p53 levels of n=3 corresponding to Figure 4G (t-test, *$P < 0.05$; *** $P < 0.001$).
M) Supplementary data Western blot gel images corresponding to Figure 4H.
N) Graphs represent p-p53 and p53 levels of n=3 corresponding to Figure 4H (t-test, * $P < 0.05$).

I

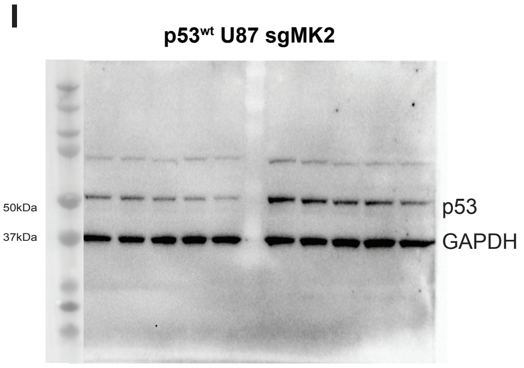
p53^{wt} U87 sgMK2
50kDa
37kDa
p53
GAPDH

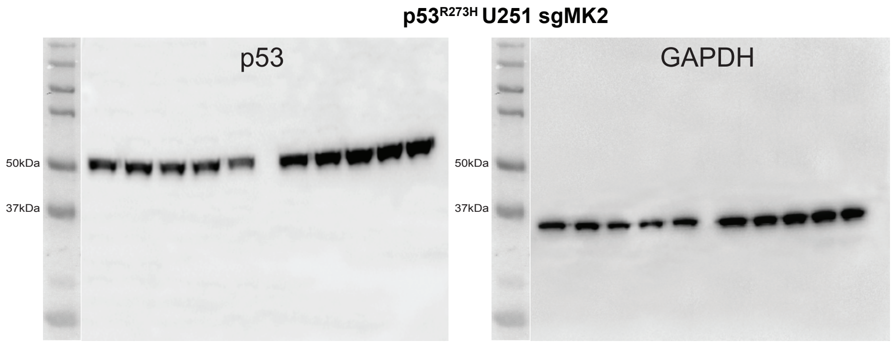
p53^{R273H} U251 sgMK2
p53
GAPDH
50kDa
37kDa

J

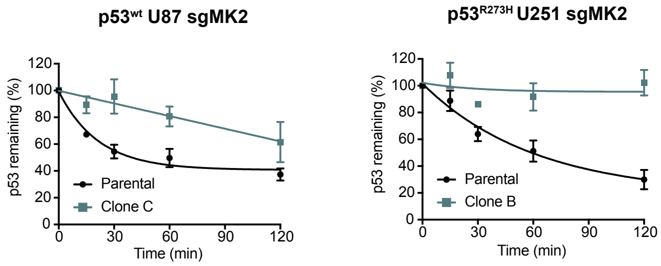
p53^{wt} U87 sgMK2
p53 remaining (%)
Time (min)
Parental
Clone C
p53^{R273H} U251 sgMK2
p53 remaining (%)
Time (min)
Parental
Clone B

K

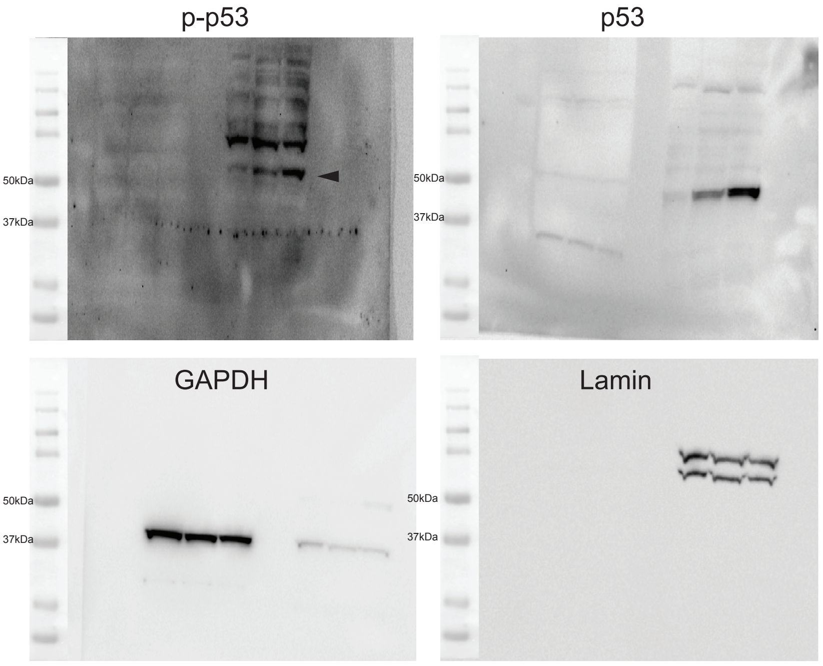
p-p53
p53
GAPDH
Lamin
50kDa
37kDa

L

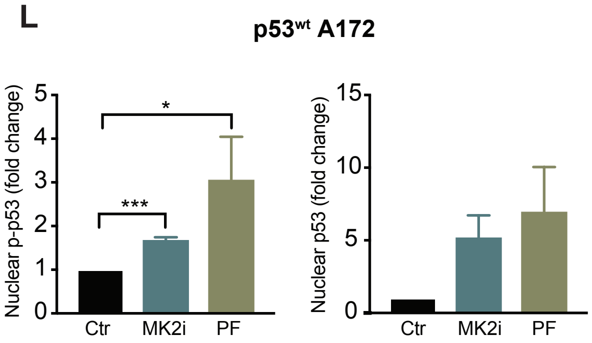
p53^{wt} A172
Nuclear p-p53 (fold change)
Nuclear p53 (fold change)
Ctr
MK2i
PF
*
***

Supplementary Figure S7

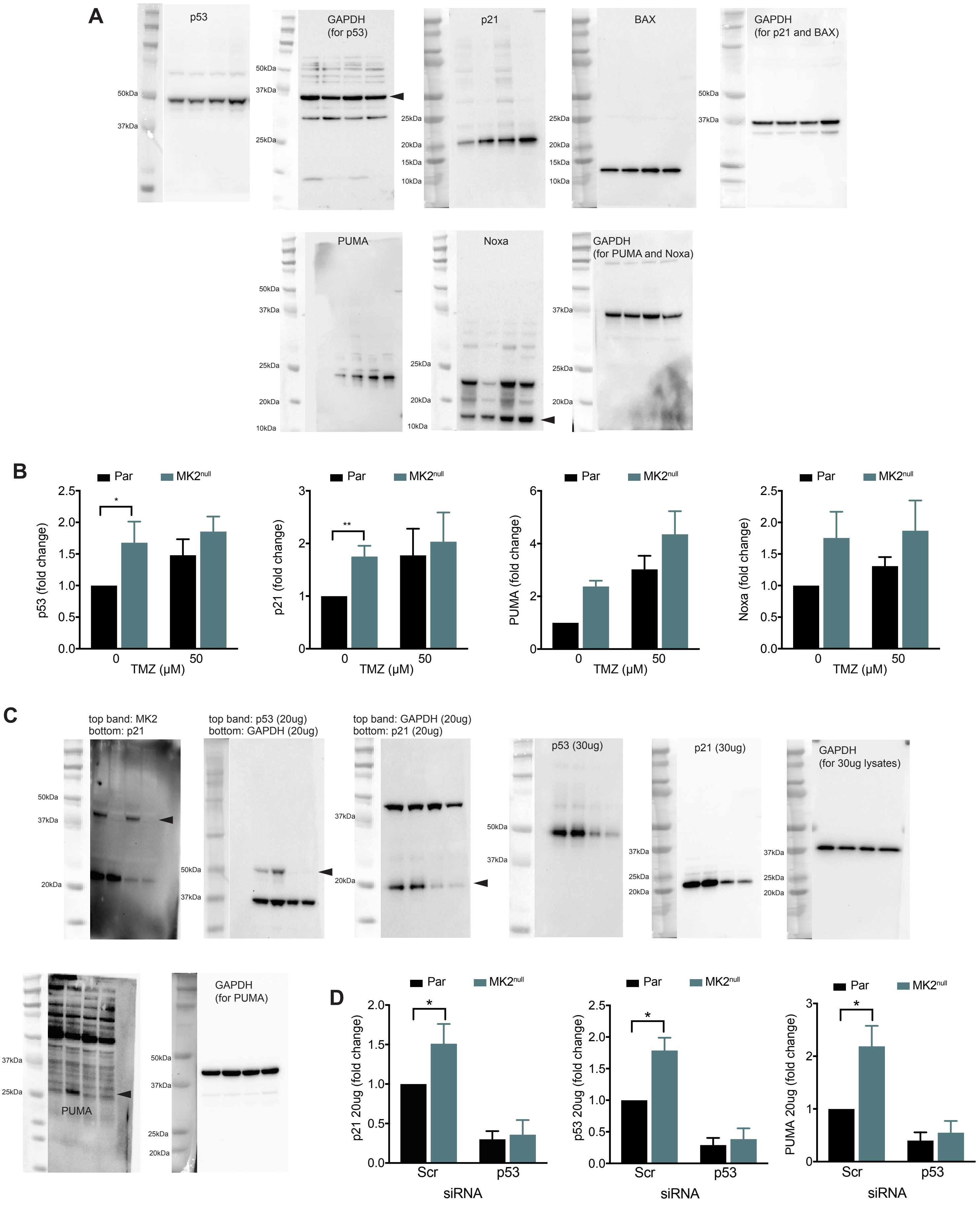

Supplementary Figure S7

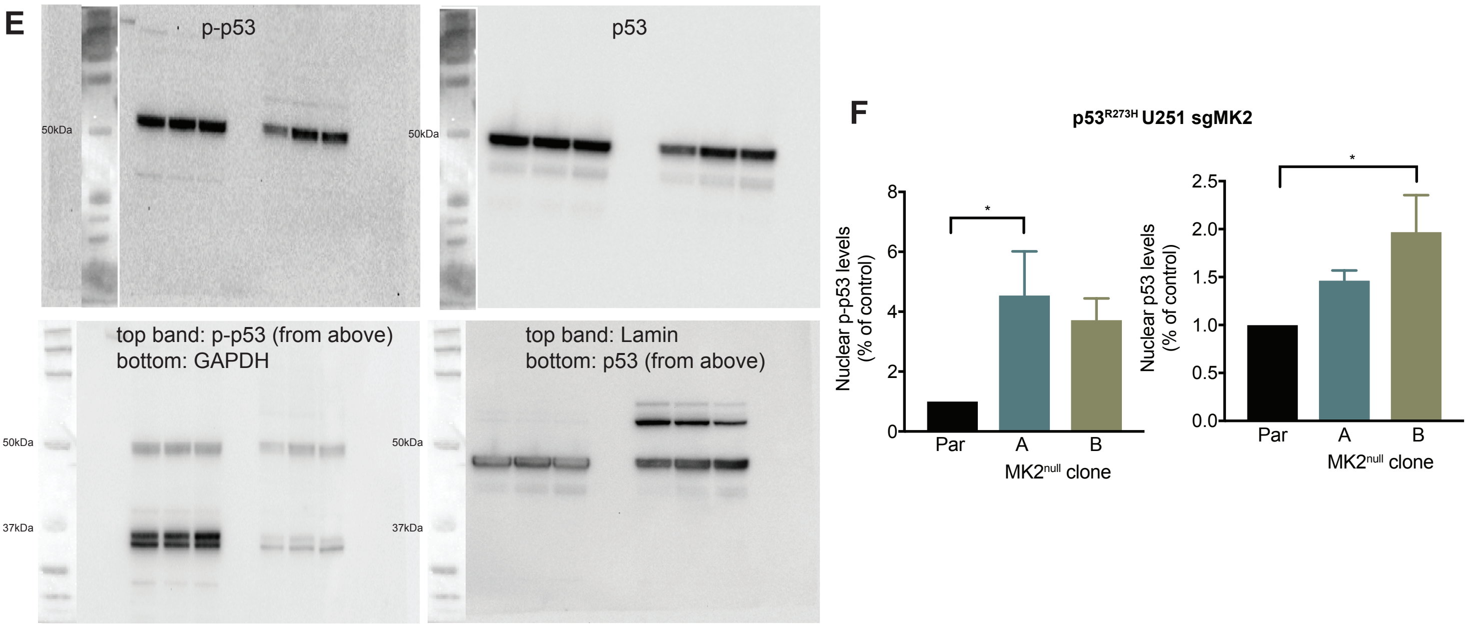

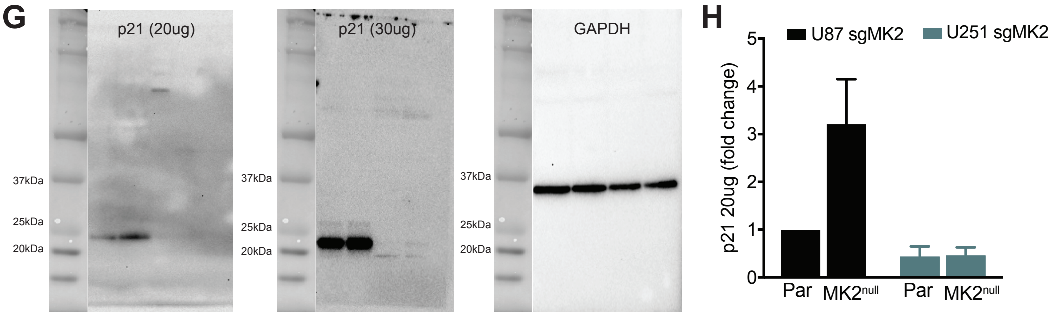

Supplementary Figure S7.

A) Supplementary data Western blot gel images corresponding to Figure 5E.

B) Graphs represent p53, p21, PUMA and Noxa levels of n=3 corresponding to Figure 5E (t-test, *P < 0.05; **P < 0.01).

C) Supplementary data Western blot gel images corresponding to Figure 5F.

D) Graphs represent p53, p21 and PUMA levels of n=3 corresponding to Figure 5F (t-test, *P < 0.05).

E) Supplementary data Western blot gel images corresponding to Figure 5H.

F) Graphs represent p-p53 and p53 levels of n=3 corresponding to Figure 5H (t-test, *P < 0.05).

G) Supplementary data Western blot gel images corresponding to Figure 5I.

H) Graph represents p21 levels of n=3 corresponding to Figure 5I.

Supplementary Figure S8

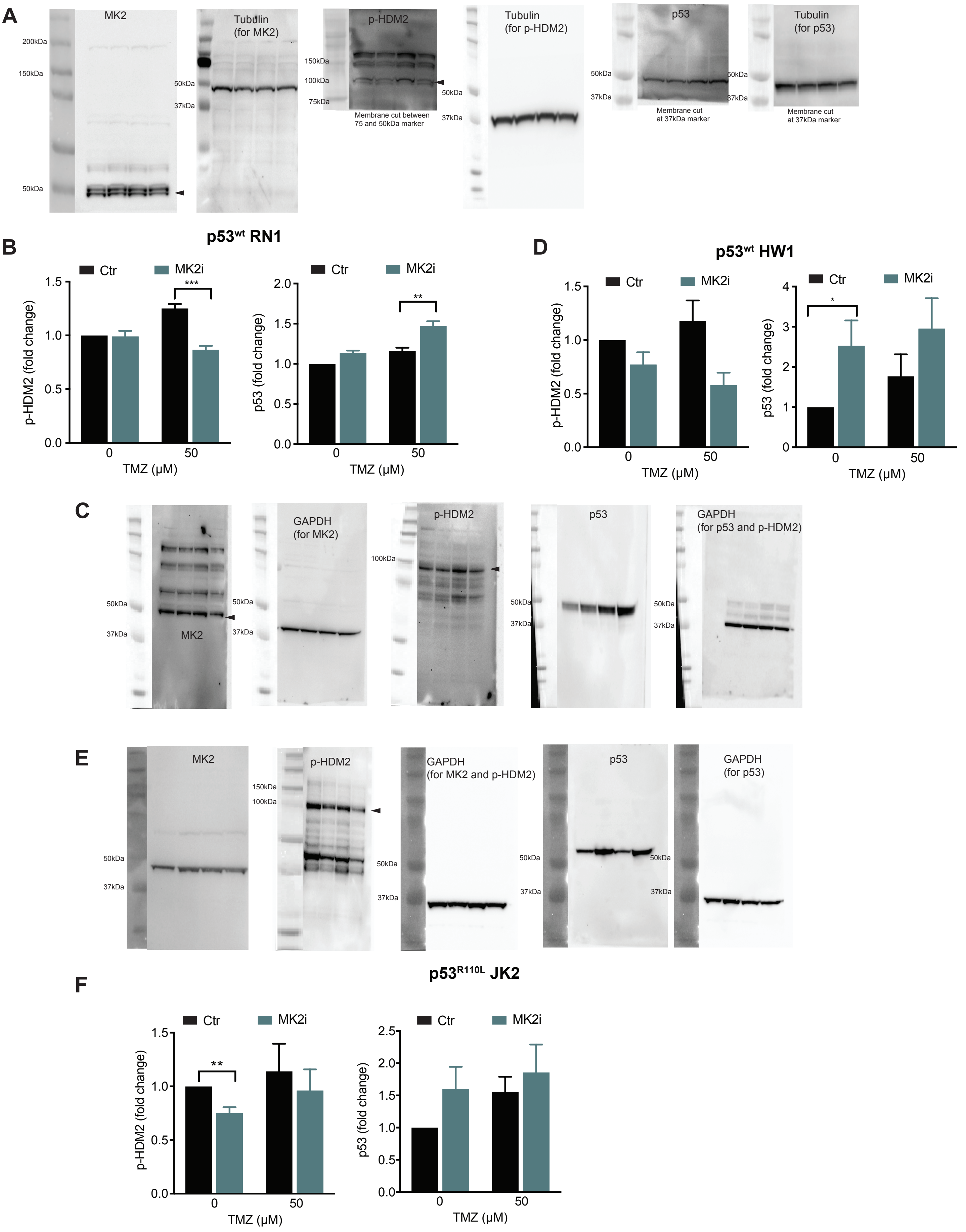

Supplemnatry Figure S8. A) Supplementary data Western blot gel images corresponding to Figure 6B.
B) Graphs represents p-HDM2 and p53 levels of n=3 corresponding to Figure 6B (t-test, **$P < 0.01$; ***$P < 0.001$).
C) Supplementary data Western blot gel images corresponding to Figure 6C.
D) Graphs represents p-HDM2 and p53 levels of n=3 corresponding to Figure 6C (t-test, *$P < 0.05$).
E) Supplementary data Western blot gel images corresponding to Figure 6H.
F) Graphs represents p-HDM2 and p53 levels of n=3 corresponding to Figure 6H (t-test, **$P < 0.01$).

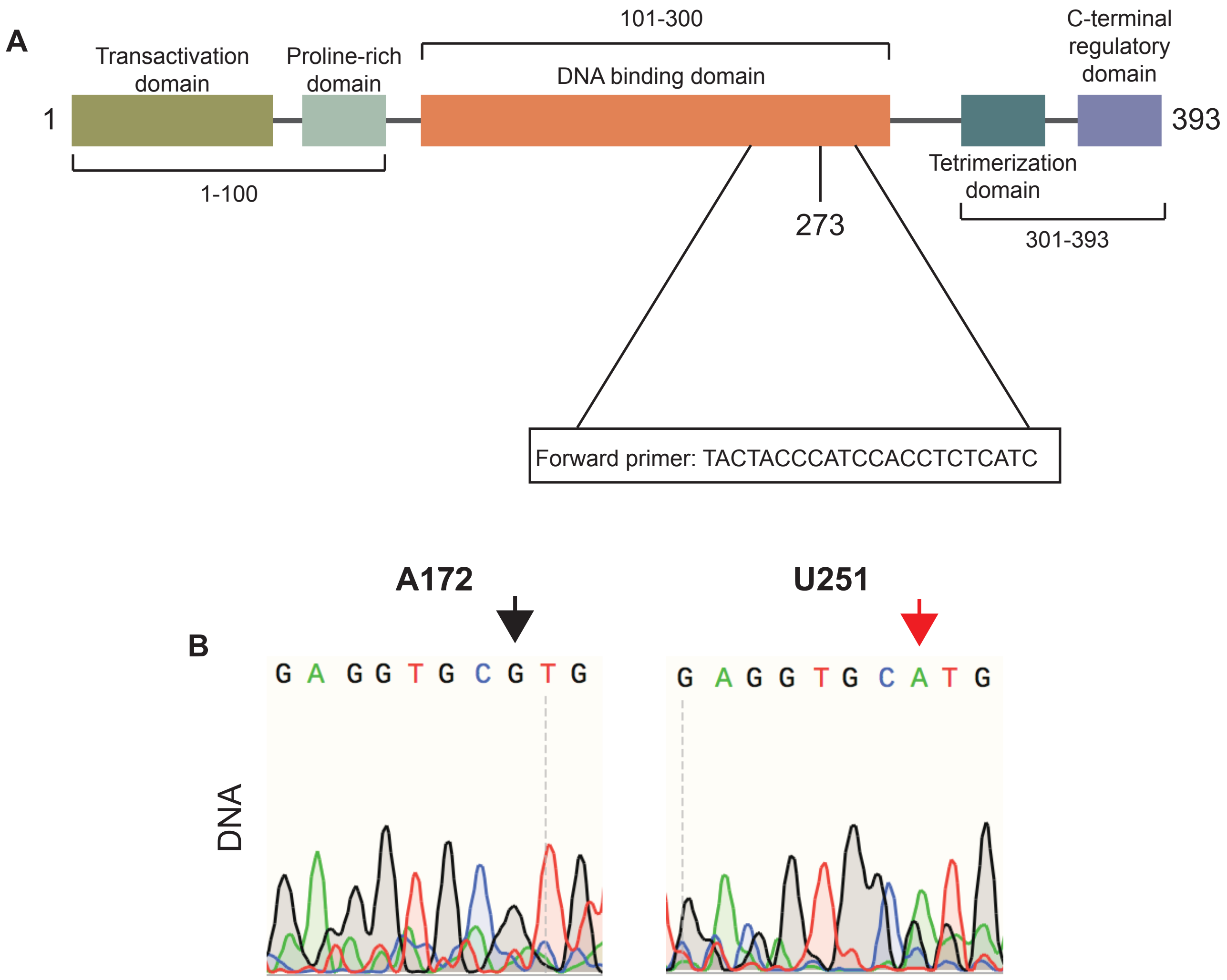

Supplementary Figure S9. A) PCR strategy used to amplify target area of TP53 for sequencing. Primers amplify a 400 bp product that contains the mutation site. Sequence of the forward primer provided. B) Chromatogram of TP53 R273H mutation. Arrow indicates base that undergoes alteration. Wild-type base being Guanine and the mutant base is Adenine.
